# Using Respiratory Sinus Arrhythmia to Estimate Inspired Tidal Volume in the Bottlenose Dolphin (*Tursiops truncatus*)

**DOI:** 10.3389/fphys.2019.00128

**Published:** 2019-02-19

**Authors:** Fabien Cauture, Blair Sterba-Boatwright, Julie Rocho-Levine, Craig Harms, Stefan Miedler, Andreas Fahlman

**Affiliations:** ^1^Departamento de Investigación, Fundación Oceanogràfic de la Comunidad Valenciana, Valencia, Spain; ^2^Department of Mathematics and Statistics, Texas A&M University–Corpus Christi, Corpus Christi, TX, United States; ^3^Dolphin Quest, Honolulu, HI, United States; ^4^Center for Marine Sciences and Technology, Department of Clinical Sciences, College of Veterinary Medicine, North Carolina State University, Morehead City, NC, United States; ^5^Veterinary Cardiology, Alboraya, Spain; ^6^Research Group on Biomedical Imaging (GIBI2^30^), Instituto de Investigación Sanitaria La Fe, Valencia, Spain

**Keywords:** electrocardiogram, spirometry, marine mammals, diving physiology, cardiorespiratory

## Abstract

Man-made environmental change may have significant impact on apex predators, like marine mammals. Thus, it is important to assess the physiological boundaries for survival in these species, and assess how climate change may affect foraging efficiency and the limits for survival. In the current study, we investigated whether the respiratory sinus arrhythmia (RSA) could estimate tidal volume (*V*_T_) in resting bottlenose dolphins (*Tursiops truncatus*). For this purpose, we measured respiratory flow and electrocardiogram (ECG) in five adult bottlenose dolphins at rest while breathing voluntarily. Initially, an exponential decay function, using three parameters (baseline heart rate, the change in heart rate following a breath, and an exponential decay constant) was used to describe the temporal change in instantaneous heart rate following a breath. The three descriptors, in addition to body mass, were used to develop a Generalized Additive Model (GAM) to predict the inspired tidal volume (*V*_Tinsp_). The GAM allowed us to predict *V*_Tinsp_ with an average ( ± SD) overestimate of 3 ± 2%. A jackknife sensitivity analysis, where 4 of the five dolphins were used to fit the GAM and the 5th dolphin used to make predictions resulted in an average overestimate of 2 ± 10%. Future studies should be used to assess whether similar relationships exist in active animals, allowing *V*_T_ to be studied in free-ranging animals provided that heart rate can be measured.

## Introduction

Marine mammals forage underwater to obtain food and therefore divide their time at the surface to exchange gasses (O_2_ and CO_2_) with submersions to different depth and of varying durations. Therefore, a better understanding of the metabolic costs associated with underwater foraging, and proxies to assess energy use would help determine how environmental change may alter survival. By increasing the duration underwater, the opportunity to encounter and obtain food, and thereby the foraging efficiency, should be increased. Man-made environmental change such as over-fishing and global warming could cause changes in prey diversity, availability and location (Perry et al., 2005), which may have detrimental effects on apex marine predators like dolphins. Changes in prey type, abundance, and distribution could result in increases in both foraging duration and distance in order to obtain enough prey for survival. Overfishing will reduce the probability to encounter food, and movement of prey to deeper depths due to ocean warming will increase the transit time and reduce the available time at the prey patch. Longer foraging bouts, and/or deeper dives may reduce the foraging efficiency and thereby cause challenges to obtain sufficient food for survival (Perry et al., 2005). Thus, understanding the cardiorespiratory traits required by marine mammals to manage life in an extreme environment, the physiological constraints imposed on these animals, and how these limitations may affect physiology and survival are crucial.

When studying animals in the wild, measuring the metabolic cost directly is challenging, and a number of proxies have been proposed and tested. One method is to measure the resting metabolic rate (RMR) by measuring the O_2_ consumption rate (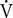
O_2_) during rest (Williams et al., 1993; Yazdi et al., 1999; Kastelein et al., 2000; Yeates and Houser, 2008; Noren et al., 2013; Rechsteiner et al., 2013; Worthy et al., 2013; van der Hoop et al., 2014; Fahlman et al., 2015), and a few studies have determined the diving and foraging metabolic rate of marine mammals during quasi-natural conditions (Kooyman et al., 1973; Sparling and Fedak, 2004; Fahlman et al., 2008, 2013). While RMR may not accurately reflect field metabolic rate (FMR), it provides an index about the minimal metabolic requirements of an individual or population against which FMR can be scaled (Bejarano et al., 2017). One method to scale FMR is to estimate FMR by validated metabolic proxies, such as heart rate (*f*_H_) (Young et al., 2011), activity (Enstipp et al., 2011; Fahlman et al., 2013), or respiratory frequency (*f*_R_) (Fahlman et al., 2016, 2017b; Folkow and Blix, 2017). Combining these methods, the metabolic costs for different populations and activities, such as resting, traveling, and foraging, can be defined. The Fick principle states that:
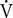
O_2_ = *f*_R_ ×*V*_T_ × (ΔO_2_), where *V*_T_ is tidal volume and ΔO_2_ the O_2_ extracted from the air inhaled with each breath. By assuming that *V*_T_ and ΔO_2_ are constant at steady state, it should be possible to estimate 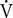
O_2_ from *f*_R_ (Folkow and Blix, 1992; Christiansen et al., 2014; Fahlman et al., 2016). While marine mammals are at the surface, *f*_R_ can be assessed during focal observations. However, this is not practical during long periods at sea. In addition, studies have shown that both *V*_T_ and ΔO_2_ change for different activities or during recovery from exercise (Fahlman et al., 2016, 2017b; Folkow and Blix, 2017), so the estimated 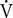
O_2_ could be improved by also estimating *V*_T_ and ΔO_2_. Consequently, methods to assess pattern of breathing (*f*_R_, *V*_T_) would provide significant advances to estimate FMR in marine mammals.

Proxies to estimate FMR from breaths should accurately predict *f*_R_ and *V*_T_ during continuous recording from free ranging animals (Fahlman et al., 2016; Rojano-Doñate et al., 2018). Such data would allow an assessment of how changes in foraging effort (duration, activity, etc.) alter respiratory function, and estimated FMR. A number of studies have assessed lung function in marine mammals under human care (Olsen et al., 1969; Kerem et al., 1975; Matthews, 1977; Kooyman and Cornell, 1981; Fahlman et al., 2015, 2018a,b, 2019; Fahlman and Madigan, 2016), and at least in the bottlenose dolphin (*Tursiops truncatus*) these data are representative of their wild counterparts, in both shallow and deep diving ecotypes (Fahlman et al., 2018a,b). Such data are important to establish baseline lung function from animals with known health under controlled situations, and provide methods that will allow proxies to be validated that can predict respiratory effort in free ranging animals.

Estimating lung function of wild populations remains difficult. One alternative proxy could be to use the changes in *f*_H_ associated with each breath, the Respiratory Sinus Arrhythmia (RSA) (de Burgh Daly, 1986). While RSA is universally present in a number of air-breathing vertebrates such as the toad, horse, dog, seal, and dolphin (Scholander, 1940; Hayano et al., 1996; Cooper et al., 2003; Noren et al., 2004; Harms et al., 2013; Zena et al., 2017; McDonald et al., 2018; Yaw et al., 2018; Piccione et al., 2019), and even in air-breathing fish (Grossman and Taylor, 2007), its physiological significance is debated (Hayano et al., 1996; Yasuma and Hayano, 2004). It has been suggested that RSA improves gas exchange by enhancing the ventilation-perfusion matching and reduces cardiac work (Yasuma and Hayano, 2004; Ben-Tal et al., 2012, 2014). The RSA causes *f*_H_ acceleration during inspiration, and deceleration during expiration (Mortola et al., 2015). Thus, continuous recordings of *f*_H_ could allow detection of *f*_R_, which when appropriately validated provide ways to estimate field metabolic rate (Fahlman et al., 2016; Rojano-Doñate et al., 2018). Considering recent progress in the development of biologging system that allow continuous recording of the electrocardiogram (ECG) in free-ranging cetaceans (Elmegaard et al., 2016; McDonald et al., 2018), we speculated that RSA may provide a novel method to estimate *V*_T_ in bottlenose dolphins. Currently, there is limited availability of commercial data loggers that can measure continuous ECG, and custom built devices range from units with implantable electrodes used in pinnipeds or diving birds (Thompson and Fedak, 1993; Woakes et al., 1995; McDonald and Ponganis, 2014), to those that are attached externally using suction cups (Noren et al., 2004; Elmegaard et al., 2016).

In the current study, we tested the hypothesis that RSA can estimate *V*_Tinsp_ in resting bottlenose dolphins by recording *f*_H_ and respiratory flow while resting at the surface. Our results provide evidence that using the RSA as a proxy allows us to estimate the average *V*_Tinsp_ of individual dolphins with an average (±SD) overestimation of 2 ± 10% with the data recorded.

## Materials and Methods

### Animals

The study protocols were approved by the Animal Care and Welfare Committee of the Oceanogràfic Foundation (OCE-17-16 and amendment OCE-29-18). Five adult male bottlenose dolphins (*T. truncatus*), housed at Dolphin Quest – Oahu (Honolulu, HI, United States), were used for all the experiments (Table 1). All experiments were conducted in January 2018. The dolphins were not restrained and could end the trial at any point. Prior to initiating the study, the dolphins were desensitized to the equipment and trained for novel research-associated behaviors using operant conditioning. Each trial consisted of the animal staying stationary in the water, allowing placement of the equipment. The animals were breathing while continuous measurements were made. Because of familiarity with these procedures, we assumed that the experimental data collected on lung function (respiratory flow) and *f*_H_ were representative of a relaxed physiological state.

**Table 1:** Dolphin ID, body mass (*M*_b_), total number of breaths analyzed (N), average ( ± SD) tidal volume (*V*_Tinsp_), and *V*_Tinsp_ range.

Dolphin ID	*M*_b_ (kg)	*N*	*V*_Tinsp_ (l)	*V*_Tinsp_ range (l)
9FL3	235.4	73	3.6 ± 1.0	1.6 - 6.2
01L5	154.6	91	3.2 ± 0.5	2.1 - 4.2
83H1	139.6	53	3.3 ± 0.8	1.8 - 5.6
9ON6	184.1	53	3.9 ± 0.6	2.7 - 5.6
6JK5	206.8	27	4.9 ± 1.2	2.7 - 6.9


### Data Acquisition

A custom-made Fleisch type pneumotachometer (Mellow Design, Valencia) utilizing a low-resistance laminar flow matrix (Item # Z9A887-2, Merriam Process Technologies, Cleveland, OH, United States) was placed over the blow-hole of the dolphin (Fahlman et al., 2015). Differential pressure across the flow matrix was measured using a differential pressure transducer (ML311 Spirometer Pod, ADInstruments, Colorado Springs, CO, United States), connected to the pneumotachometer with two, 310 cm lengths of 2 mm I.D., firm walled, flexible tubing. The pneumotachometer was calibrated using a 7.0 l calibration syringe (Series 4900, Hans-Rudolph Inc., Shawnee, KS, United States). The signal was integrated and the flow determined assuming a linear response between differential pressure and flow. The linear response of the pneumotachometer was confirmed by calibrating with the 7.0 l syringe immediately before and after each trial, through a series of pump cycles at various flows. The pump cycles allowed the relationship between differential pressure and flows for the expiratory and inspiratory phases to be determined. All gas volumes were converted to standard temperature pressure dry (STPD) (Quanjer et al., 1993). Exhaled air was assumed saturated at 37°C, inhaled air volume was corrected for ambient temperature and relative humidity, and *V_T_* was calculated by integrating the flow as previously detailed (Fahlman et al., 2015).

The electrocardiogram (ECG) was recorded using three gold-plated electrodes mounted inside a silicone suction cup connected to a custom-built data recorder (UUB/1-ECGb, UFI, Morro Bay, CA, United States). The three electrodes were placed on the ventral surface: red on the right side close to the pectoral fin, yellow opposite on the left side, and green on the right side approximately 30 cm more caudally from the red. The suctions cups were filled with conducting gel (Redux Gel, Parker Laboratories) before being placed on the skin. Next, the animal rolled over to ensure the suction cups stayed in place.

The respiratory flow and ECG were recorded at 400 Hz using a data acquisition system (Powerlab 8/35, ADInstruments, Colorado Springs, CO, United States), and displayed on a computer running LabChart (v. 8.1, ADInstruments, Colorado Springs, CO, United States). Initially, the electrodes were adjusted to assure a clear ECG trace. Next, the pneumotachometer was placed over the blow-hole and the animal allowed to breathe spontaneously for up to 10 min.

We used the ECG analysis routine in LabChart to automatically detect the time between R-R peaks using the following settings; typical QRS width = 80 ms, R-waves = 300 ms, pre-P baseline = 120 ms, maximum PR = 240 ms, maximum RT = 400 ms. The detected R peaks were then manually verified and the instantaneous heart rate (i*f*_H_) determined from the time between R-R peaks.

### Data Processing, Statistical Analysis and Modeling

All data were analyzed using R (version 3.4.3 – © 2017 The R Foundation for Statistical Computing) through RStudio (version 1.1.383 – © 2009–2017 RStudio, Inc.). Initially the temporal changes in i*f*_H_ were described for each breath. We used a function that fit the exponential decay with time following the beginning of the inspiration for each breath:

(1) $$\text{i}f_{H}\text{=}\;\text{Base}\;\text{Heart}\;\text{Rate}\;\text{+}\;{\text{e}}^{\text{-Decay}\;\text{rate}\times \text{Time}}\times \text{Initial change in heart rate}$$

Equation 1 was fit for each breath using the “L-BFGS-B” method of the “optim” function (Byrd et al., 1995), which optimizes parameters between imposed bounds to restrain parameters to physiologically relevant values. Breaths with fewer than seven beats after the inhalation were excluded (44 breaths).

Next the three parameters from Eq. 1 (Base Heart Rate, Decay rate, and initial change in heart rate) for each breath, and body mass (*M*_b_) were fit against inhaled *V*_T_ (*V*_Tinsp_) using a loess Generalized Additive Model (GAM) (Cleveland, 1979; Hastie and Tibshirani, 1990), with the span fixed at 0.34.

To assess the sensitivity of the model, we generated five different GAMs by excluding all observations from one dolphin each time. The data from the excluded dolphin was then used to predict *V*_Tinsp_. The error was computed using the formula:

(2) $$\text{Prediction}\;\text{error=}\;\frac{\left(\text{Predicted-Measured}\right)}{\text{Measured}}\;\times \left(\text{-100}\right)\;\;(2)$$

where a positive value represents an overestimated prediction.

## Results

### Data Used for the Analysis

A total of 297 breaths were analyzed following removal of breaths with less than seven heart beats between breaths (Table 1). Only spontaneous breaths were used for the analysis, which limited the range of *V*_T_’s. In addition, as not all inspired and expired volumes are similar for each breath, we only used the *V*_Tinsp_ for the analysis.

The average (±SD) *V*_Tinsp_ was 3.8 ± 0.7 l (range: 1.6–6.9 l, see Table 1 for individual variation), and the average duration between breaths was 15.3 ± 10.7 s (range: 4–129 s). The average i*f*_H_ was 74 ± 24 beats min^-1^ (range: 27–293 beats **⋅** min^-1^). The average fit parameters for Equation 1 for each dolphin are reported in Table 2.

**Table 2:** Dolphin ID, average fit parameters for Equation 1 [base heart rate (*f*_H_), decay, initial jump (Δ*f*_H_)], and average inspired tidal volume (*V*_Tinsp_).

Dolphin ID	Base *f*_H_	Decay	Δ*f*_H_	Average *V*_Tinsp_
9FL3	34.1	0.0362	37.2	3.6
01L5	37.9	0.0486	46.2	3.2
83H1	39.7	0.0523	46.8	3.3
9ON6	41.2	0.0311	50.5	3.9
6JK5	48.6	0.0280	49.2	4.9
Mean (±SD)	40.3 ± 5.4	0.0393 ± 0.0108	46.0 ± 5.2	3.8 ± 0.7


### Predicting *V*_T_ From Instantaneous *f*_H_

**FIGURE 1:**
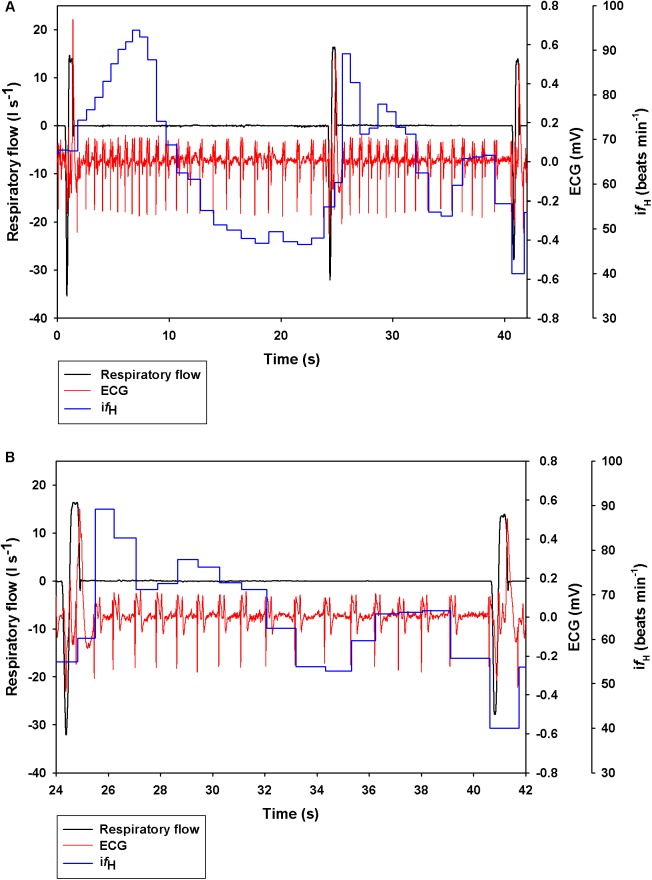
Representative data showing respiratory flow, ECG, and instantaneous heart rate (i*f*_H_) in a bottlenose dolphin during **(A)** 3 breaths, or **(B)** zoomed in for the 2nd breath.

**FIGURE 2:**
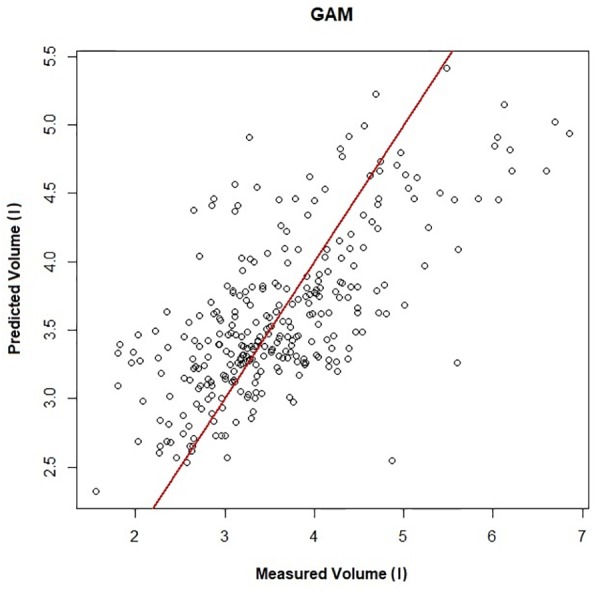
Predicted vs. measured inspired tidal volume (*r*^2^ = 0.45). The GAMs model is used to generate the predicted volume, and measured volume is the inspired tidal volume measured using the pneumotachometer, red line is the line of unity.

**FIGURE 3:**
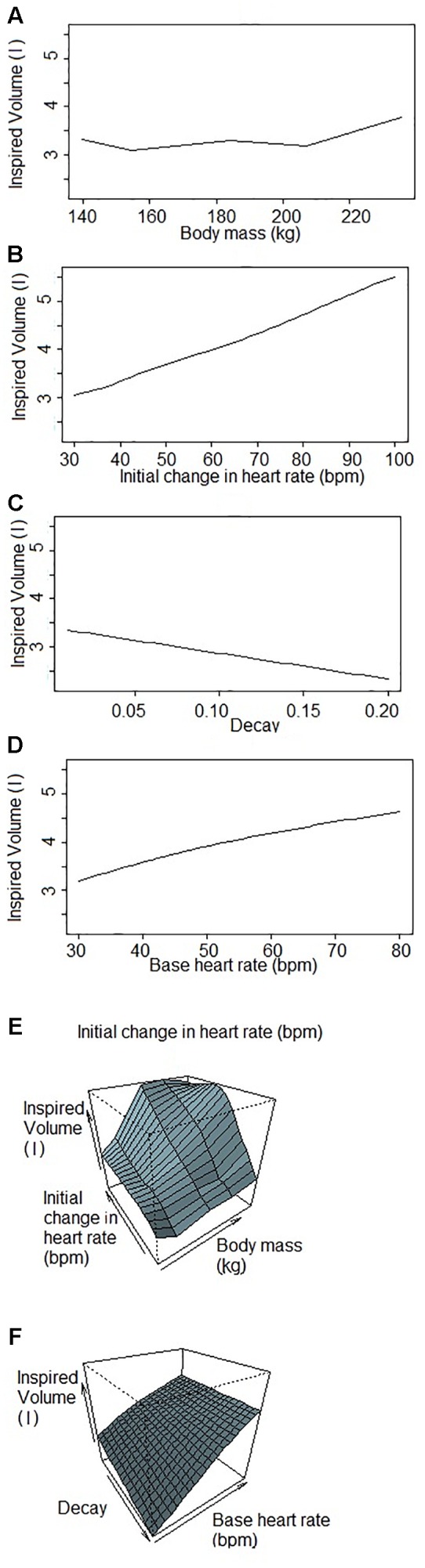
Sensitivity analysis of each variable used to predict inspired tidal volume by the Generalized Additive Model when one (or two) factor(s) changes while others are fixed. Inspired volumes are in liters. **(A)** Inspired volume as a function of body mass. **(B)** Inspired volume as a function of the initial change in heart rate. The initial jump is the parameter of the GAM that explains the most variation in inspired volume of the four parameters. **(C)** Inspired volume as a function of the decay. The decay is the parameter that explains the lowest variation in the GAM. **(D)** Inspired volume as a function of the base heart rate. The base heart rate is the variable that has the second most influence on the inspired volume predicted by the GAM. **(E,F)** Inspired volume as a function of two parameters **(E)** body mass and initial change in heart rate; **(F)** decay and base heart rate. These figures illustrate the covariance of the parameters that have consequences for the predicted inspired volume.

Figure 1 shows a representative ECG trace, i*f*_H_, and respiratory flow in a dolphin over 3 breaths. The average conditions for estimating *V*_Tinsp_ are reported in Table 2, and the GAMs overestimated *V*_Tinsp_ by an average 3 ± 2% (range of individual average error: 0.3 to 7.4%, Figure 2). A sensitivity analysis was performed to assess how the prediction changed with changes in each variable (Figure 3). The decay rate and *M*_b_ had less influence on the model output as compared with base *f*_H_ and the initial change in *f*_H_.

**FIGURE 4:**
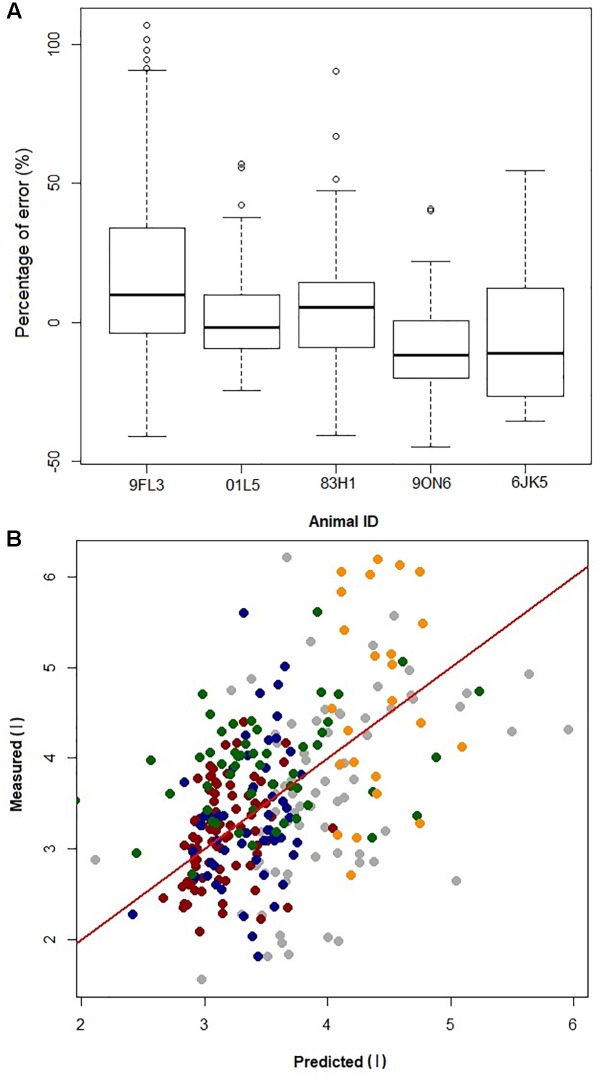
**(A)** Boxplot of prediction error [error = (predicted–measured)/ measured × 100] from jackknife sensitivity analysis, where the data from one dolphin (Animal ID) is removed to generate the GAMs and the resulting GAMs model is used to predict *V*_Tinsp_ for that dolphin. **(B)** Plot of error in prediction of a single dolphin *V*_T_ when building the GAM using data from the other four dolphins. Gray = 9FL3; Red = 01L5; Blue = 83H1; Green = 9ON6; Orange = 6JK5; Red line is identity line.

By removing one dolphin, fitting the GAMs with the four dolphins, and then predicting *V*_Tinsp_ for the 5th dolphin resulted in an average (±SD) overestimation of 2 ± 10%, (range of individual average error: -10 to 18%, Figure 4A,B). The error for individual breaths ranged from 107 to -45%, with 95% confidence limits ranging between 12 to -7% (median: 12 to -10%, Figure 4B).

## Discussion

The main objective with the current study was to determine if the changes in *f*_H_ associated with RSA can be used to predict the *V*_Tinsp_ in the bottlenose dolphin. For this purpose, we collected continuous ECG and respiratory flow in bottlenose dolphins. A jackknife method to resample the data showed that RSA, in addition to *M*_b_, can be used to predict the average *V*_Tinsp_ of an individual dolphin to within 2 ± 10% of the measured value, and all individual average prediction errors were less than 20%. This shows that the GAMs should be able to predict the average *V*_Tinsp_ of individual dolphins using data from another population of bottlenose dolphins. If future studies can verify a similar relationship in active animals, RSA could be a useful proxy to estimate *V*_Tinsp_ from free-ranging marine mammals as methods to continuously measure *f*_H_ are developed.

The average i*f*_H_ reported in the current study was similar to those reported in previous studies in the bottlenose dolphin (ranging from 60 to 105 beats min^-1^) (Noren et al., 2004, 2012; Houser et al., 2010), when the *f*_H_ is calculated without accounting for the RSA. However, in our past study, using trans-thoracic echocardiography to measure *f*_H_ and stroke volume, it was pointed out that estimating *f*_H_ without accounting for the RSA will result in average surface *f*_H_ values that are confounded by the *f*_R_ (Miedler et al., 2015). This is particularly problematic in marine mammals, with an *f*_R_ ranging from 1 to 5 breaths **⋅** min^-1^ (Piscitelli et al., 2013; Fahlman et al., 2017a). Consequently, estimating resting *f*_H_ without accounting for the RSA will overestimate the resting *f*_H_. As these resting *f*_H_’s have been used to assess the magnitude of the cardiovascular changes associated with diving they would erroneously overestimate the magnitude of the dive response (Fahlman et al., unpublished). The average base *f*_H_ in the current study (Table 2, 40 ± 5 beats min^-1^) was similar to those reported in our past study in the bottlenose dolphin (*f*_H_ = 41 ± 9 beats min^-1^) (Miedler et al., 2015), where the RSA was accounted for. Consequently, the base *f*_H_ reported in the current study is a more appropriate value for the resting *f*_H_ in the bottlenose dolphin. If this value is used, it provides an interesting perspective as that value is similar to the diving bradycardia reported in previous studies (Noren et al., 2012). Thus, we propose that future studies should evaluate the resting *f*_H_ in voluntary diving animals after correcting for the RSA.

While the current method clearly shows that RSA is useful to estimate *V*_Tinsp,_ there are a number of limitations with the current method. First of all, due to the limited data set, we aimed to reduce the number of parameters used in the model. To simplify the analysis the current method did not include the duration between breaths. We analyzed each breath separately, which was both time consuming and does not account for the dependence between breaths. However, breathing and *f*_H_ are continuous data. Future studies could assess time-series methods to predict *V*_Tinsp_, which allows the dependence between breaths to be considered. For Equation 1, there was considerable variation in the fitted values for the base *f*_H_. When accounting for the changes in *f*_H_ associated with a breath there is usually minimal variation in the base *f*_H_ within one dolphin (Miedler et al., 2015). The large variation could be related to varying duration between breaths, which may alter the base *f*_H_. Thus, for breaths close together the *f*_H_ may not have reached the base *f*_H_ before the next breath, and may have influenced the *f*_H_ variation for the next breath. Based on the current analysis, this method cannot accurately predict the *V*_Tinsp_ of individual breaths, but was able to provide reliable average estimated *V*_Tinsp_ for each animal based on the GAMs fitted to the other animals. This is similar to the method using *f*_H_ to estimate field metabolic rate, where the there are limitations to estimate the metabolic cost for each dive, but where large data sets are able to estimate the energy requirements for different activities (Fahlman et al., 2004; Halsey et al., 2007; Young et al., 2011). Given the limitations with the current data, we propose that further development of this method may provide an interesting approach to study cardiorespiratory physiology in free-ranging marine mammals.

The *V*_Tinsp_ in the current study were of limited range (average ± SD = 3.8 ± 0.7 l, range: 1.6–6.9 l). While variation in the *V*_Tinsp_ during voluntary breaths is difficult to control, animals under managed care can be trained to perform maximal respiratory effort, which allows *V*_T_ to vary over a much greater range (Kooyman and Cornell, 1981; Fahlman et al., 2015). For example, maximal respiratory efforts in dolphins in the weight range of the current study would increase the *V*_T_ range to around 20 l (Kooyman and Cornell, 1981; Fahlman et al., 2015). It would also be useful to include females and individuals from other age classes to increase the range of variation and allow the model to be used for wild dolphin populations.

In addition, future studies should also assess whether this method is robust enough to study active or free-ranging dolphins. During natural dives *f*_R_ and *V*_T_ may both be highly variable and irregular, and alterations in the relationship between RSA, vagal tone, *f*_R_, and *V*_T_ (de Burgh Daly, 1986; Ben-Tal et al., 2014; Guillén-Mandujano and Carrasco-Sosa, 2014; Mortola et al., 2016) may limit the accuracy of a model developed for dolphins at rest. Thus, changes in activity state, e.g., exercise, rest, travel, diving, may significantly influence autonomic tone and alter the relationship.

Finally, the GAM model does not provide an estimate of uncertainty around the predicted values with new data (data that were not in the dataset used to fit the model), nor does it provide a prediction equation for the non-parametric part of the model. To avoid these drawbacks, additional measurements with a larger range of *V*_Tinsp_, *f*_R_, and activity states would help define a prediction equation that could be used in free-ranging dolphins. In addition, we propose that this method could be used for other cetaceans and marine mammal species that exhibit significant RSA. If future studies are able to verify that this method is able to estimate *f*_R_ and *V*_Tinsp_ in actively swimming or diving dolphins this method may provide a predictive procedure for free-ranging mammals that may significantly enhance our knowledge of how marine mammals partition energy use during diving, and how the environment may limit foraging efficiency.

In summary, we show that that RSA can be used to accurately predict the average *V*_Tinsp_ of individual resting bottlenose dolphins with an average overestimated error of 2 ± 10%. While a number of factors appear to alter RSA (de Burgh Daly, 1986), the universal existence of RSA in vertebrates, and the suggestions that it is independent of body size (Piccione et al., 2019), could provide a method to study cardiorespiratory physiology in free ranging marine vertebrates, from marine mammals, to birds and reptiles, unraveling important mechanisms to understand the ecophysiology of these species.

## Data Availability Statement

The data used in this study are freely available at the following link: osf.io/buwdp.

## Author Contributions

FC performed the data and statistical analysis and drafted the first draft of the manuscript. BS-B provided statistical advice and edited the manuscript. JR-L supervised all animal training and helped with all research trials. CH helped conceive the study and helped consult with the methods. SM helped with the trials and the analysis. AF conceived the study, designed the experiments, collected the data, provided funding, helped with the data analysis, and helped to draft the manuscript. All authors helped to revise the various drafts and gave final permission to publish the study.

## Conflict of Interest Statement

The authors declare that the research was conducted in the absence of any commercial or financial relationships that could be construed as a potential conflict of interest.

## References

[B1] Bejarano A. C. Wells R. S. Costa D. P. (2017). Development of a bioenergetic model for estimating energy requirements and prey biomass consumption of the bottlenose dolphin *Tursiops truncatus*. *Ecol. Model.* 356 162–172. 10.1016/j.ecolmodel.2017.05.001

[B2] Ben-Tal A. Shamailov S. S. J. Paton F. R. (2012). Evaluating the physiological significance of respiratory sinus arrhythmia: looking beyond ventilation–perfusion efficiency. *J. Physiol.* 590 1989–2008. 10.1113/jphysiol.2011.222422 22289913 PMC3573317

[B3] Ben-Tal A. Shamailov S. S. J. Paton F. R. (2014). Central regulation of heart rate and the appearance of respiratory sinus arrhythmia: new insights from mathematical modeling. *Math. Biosci.* 255 71–82. 10.1016/j.mbs.2014.06.015 25004397 PMC4146737

[B4] Christiansen F. Rasmussen M. H. Lusseau D. (2014). Inferring energy expenditure from respiration rates in minke whales to measure the effects of whale watching boat interactions. *J. Exp. Mar. Biol. Ecol.* 459 96–104. 10.1016/j.jembe.2014.05.014

[B5] Cleveland W. S. (1979). Robust locally weighted regression and smoothing scatterplots. *J. Am. Stat. Assoc.* 74 829–836. 10.1080/01621459.1979.10481038

[B6] Cooper H. E. Parkes M. J. Clutton-Brock T. H. (2003). CO2-dependent components of sinus arrhythmia from the start of breath holding in humans. *Am. J. Physiol. Heart Circ. Physiol.* 285 H841–H848. 10.1152/ajpheart.01101.2002 12730051

[B7] de Burgh Daly M. (1986). “Interactions Between Respiration and Circulation,” in *Handbook of Physiology-the Respiratory System, Control of Breathing*, eds Cherniak N. S. Widdicombe J. G. (Bethesda, MD: American Physiological Society).

[B8] Elmegaard S. L. Johnson M. Madsen P. T. McDonald B. I. (2016). Cognitive control of heart rate in diving harbor porpoises. *Curr. Biol.* 26 R1167–R1176. 10.1016/j.cub.2016.10.020 27875692

[B9] Enstipp M. R. Ciccione S. Gineste B. Milbergue M. Ballorain K. Y. Ropert-Coudert Y. (2011). Energy expenditure of freely swimming adult green turtles (*Chelonia mydas*) and its link with body acceleration. *J. Exp. Biol.* 214 4010–4020. 10.1242/jeb.062943 22071193

[B10] Fahlman A. Brodsky M. Miedler S. Dennison S. Ivančić M. Levine G. (2019). Ventilation and gas exchange before and after voluntary static surface breath-holds in clinically healthy bottlenose dolphins, *Tursiops truncatus*. *J. Exp. Biol.* (in press). 10.1242/jeb.192211 30760549

[B11] Fahlman A. Brodsky M. Wells R. McHugh K. Allen J. Barleycorn A. (2018a). Field energetics and lung function in wild bottlenose dolphins, *Tursiops truncatus*, in sarasota bay florida. *Roy. Soc. Open. Sci.* 5:171280. 10.1098/rsos.171280 29410836 PMC5792913

[B12] Fahlman A. McHugh K. Allen J. Barleycorn A. Allen A. Sweeney J. (2018b). Resting metabolic rate and lung function in wild offshore common bottlenose dolphins, *Tursiops truncatus*, near bermuda. *Front. Physiol.* 9:886. 10.3389/fphys.2018.00886 30065656 PMC6056772

[B13] Fahlman A. Handrich Y. Woakes A. J. Bost C. A. Holder R. Duchamp C. (2004). Effect of fasting on the VO2-fh relationship in king penguins, aptenodytes patagonicus. *Am. J. Physiol.* 287 R870–R877. 15178544 10.1152/ajpregu.00651.2003

[B14] Fahlman A. Loring S. H. Levine G. Rocho-Levine J. Austin T. Brodsky M. (2015). Lung mechanics and pulmonary function testing in cetaceans *J. Exp. Biol.* 218 2030–2038. 10.1242/jeb.119149 26157159

[B15] Fahlman A. Madigan J. (2016). Respiratory function in voluntary participating patagonia sea lions in sternal recumbency. *Front. Physiol*. 7:528 10.3389/fphys.2016.00528 27899896 PMC5110536

[B16] Fahlman A. Moore M. J. Garcia-Parraga D. (2017a). Respiratory function and mechanics in pinnipeds and cetaceans. *J. Exp. Biol.* 220 1761–1763. 10.1242/jeb.126870 28515170

[B17] Fahlman A. van der Hoop J. Moore M. J. Levine G. Rocho-Levine J. Brodsky M. (2017b). Response to ‘On the importance of understanding physiology when estimating energetics in cetaceans’. *Biol. Open* 6 307–308. 10.1242/bio.023143 28202473 PMC5312109

[B18] Fahlman A. Svärd C. Rosen D. A. S. Jones D. R. Trites A. W. (2008). Metabolic costs of foraging and the management of O2 and CO2 stores in steller sea lions. *J. Exp. Biol.* 211 3573–3580. 10.1242/jeb.023655 18978221

[B19] Fahlman A. Svärd C. Rosen D. A. S. Wilson R. S. Trites A. W. (2013). Activity as a proxy to estimate metabolic rate and to partition the metabolic cost of diving vs. breathing in pre- and post-fasted Steller sea lions. *Aquat. Biol.* 18 175–184. 10.3354/ab00500

[B20] Fahlman A. van der Hoop J. Moore M. Levine G. Rocho-Levine J. Brodsky M. (2016). Estimating energetics in cetaceans from respiratory frequency: why we need to understand physiology. *Biol. Open* 15 436–442. 10.1242/bio.017251 26988759 PMC4890674

[B21] Folkow L. P. Blix A. S. (1992). Metabolic rates of minke whales (*Balaenoptera acutorostrata*) in cold water. *Acta. Physiol. Scand.* 146 141–150. 10.1111/j.1748-1716.1992.tb09402.x 1442122

[B22] Folkow L. P. Blix A. S. (2017). On the importance of understanding physiology when estimating energetics in cetaceans. *Biol. Open* 6 306–308. 10.1242/bio.023929 28202472 PMC5312112

[B23] Grossman P. Taylor E. W. (2007). Toward understanding respiratory sinus arrhythmia: relations to cardiac vagal tone, evolution and biobehavioral functions. *Biol. Psychol.* 74 263–285. 10.1016/j.biopsycho.2005.11.014 17081672

[B24] Guillén-Mandujano A. Carrasco-Sosa S. (2014). Additive effect of simultaneously varying respiratory frequency and tidal volume on respiratory sinus arrhythmia. *Auton. Neurosci.* 186 69–76. 10.1016/j.autneu.2014.08.003 25200867

[B25] Halsey L. G. Fahlman A. Handrich Y. Schmidt A. Woakes A. J. Butler P. J. (2007). How accurately can we estimate energetic costs in a marine top predator, the king penguin? *Zoology* 110 81–92. 17363231 10.1016/j.zool.2006.09.001

[B26] Harms C. A. Jensen E. D. Townsend F. I. Hansen L. J. Schwacke L. H. Rowles T. K. (2013). Electrocardiograms of bottlenose dolphins (*Tursiops truncatus*) out of water: habituated colelction versus wild postcapture animals. *J. Zoo Wildlife Med.* 44 972–981. 10.1638/2013-0093.1 24450057

[B27] Hastie T. Tibshirani R. (1990). Generalized additive models. *Stat. Sci.* 43:335. 10.1177/096228029500400302 8548102

[B28] Hayano J. Yasuma F. Okada A. Mukai S. Fujinami T. (1996). Respiratory sinus arrhythmia. a phenomenon improving pulmonary gas exchange and circulatory efficiency. *Circulation* 94 842–847. 10.1161/01.CIR.94.4.842 8772709

[B29] Houser D. S. Dankiewicz-Talmadge L. A. Stockard T. K. Ponganis P. J. (2010). Investigation of the potential for vascular bubble formation in a repetitively diving dolphin. *J. Exp. Biol.* 213 52–62. 10.1242/jeb.028365 20008362

[B30] Kastelein R. A. Mosterd J. Schooneman N. M. Wiepkema P. R. (2000). Food consumption, growth, body dimensions, and respiration rates of captive false killer whales (*Pseudorca crassidens*). *Aquat. Mam.* 26 33–44.

[B31] Kerem D. H. Kylstra J. A. Saltzman H. A. (1975). Respiratory flow rates in the sea lion. *Undersea Biomed. Res.* 2 20–27. 1181704

[B32] Kooyman G. L. Cornell L. H. (1981). Flow properties of expiration and inspiration in a trained bottle-nosed porpoise. *Physiol. Zool.* 54 55–61. 10.1086/physzool.54.1.30155804

[B33] Kooyman G. L. Kerem D. H. Campbell W. B. Wright J. J. (1973). Pulmonary gas exchange in freely diving weddell seals (*Leptonychotes weddelli*). *Resp. Physiol.* 17 283–290. 10.1016/0034-5687(73)90003-0 4702979

[B34] Matthews R. C. (1977). *Pulmonary Mechanics of California Sea Lions, Zalophus Californianus.* San Diego: San Diego State University.

[B35] McDonald B. I. Johnson M. Madsen P. T. (2018). Dive heart rate in harbour porpoises is influenced by exercise and expectations. *J. Exp. Biol.* 221:jeb168740. 10.1242/jeb.168740 29122951

[B36] McDonald B. I. Ponganis P. J. (2014). Deep-diving sea lions exhibit extreme bradycardia in long duration dives. *J. Exp. Biol.* 217 1525–1534. 10.1242/jeb.098558 24790100

[B37] Miedler S. Fahlman A. Valls Torres M. Álvaro Álvarez T. Garcia-Parraga D. (2015). Evaluating cardiac physiology through echocardiography in bottlenose dolphins: using stroke volume and cardiac output to estimate systolic left ventricular function during rest and following exercise. *J. Exp. Biol.* 218 3604–3610. 10.1242/jeb.131532 26385334

[B38] Mortola J. P. Marghescu D. Siegrist-Johnstone R. (2015). Respiratory sinus arrhythmia in young men and women at different chest wall configurations. *Clin. Sci.* 128 507–516. 10.1042/CS20140543 25387977

[B39] Mortola J. P. Marghescu D. Siegrist-Johnstone R. (2016). Thinking about breathing: effects on respiratory sinus arrhythmia. *Resp. Physiol. Neurobiol.* 223 28–36. 10.1016/j.resp.2015.12.004 26724603

[B40] Noren D. P. Holt M. M. Dunkin R. C. Williams T. M. (2013). The metabolic cost of communicative sound production in bottlenose dolphins (*Tursiops truncatus*). *J. Exp. Biol.* 216 1624–1629. 10.1242/jeb.083212 23393280

[B41] Noren S. R. Cuccurullo V. Williams T. M. (2004). The development of diving bradycardia in bottlenose dolphins (*Tursiops truncatus*). *J. Comp. Physiol. B.* 174 139–147. 10.1007/s00360-003-0398-9 14639484

[B42] Noren S. R. Kendall T. Cuccurullo V. Williams T. M. (2012). The dive response redefined: underwater behavior influences cardiac variability in freely diving dolphins. *J. Exp. Biol.* 215 2735–2741. 10.1242/jeb.069583 22837445

[B43] Olsen C. R. Hale F. C. Elsner R. (1969). Mechanics of ventilation in the pilot whale. *Resp. Physiol.* 7 137–149. 10.1016/0034-5687(69)90001-2 5823828

[B44] Perry A. L. Low P. J. Ellis J. R. Reynolds J. D. (2005). Climate change and distribution shifts in marine fishes. *Science* 308 1912–1915. 10.1126/science.1111322 15890845

[B45] Piccione G. Giudice E. Giannetto C. Mortola J. P. (2019). The magnitude of respiratory sinus arrhythmia of a large mammal (the horse) is like that of humans. *Resp. Physiol. Neurobiol.* 259 170–172. 10.1016/j.resp.2018.09.006 30240721

[B46] Piscitelli M. A. Raverty S. A. Lillie M. A. Shadwick R. E. (2013). A review of cetacean lung morphology and mechanics. *J. Morphol.* 274 1425–1440. 10.1002/jmor.20192 24027086

[B47] Quanjer P. H. Tammeling G. J. Cotes J. E. Pedersen O. F. Peslin R. Yernault J.-C. (1993). Lung volumes and forced ventilatory flows. *Euro. Resp. J.* 6 5–40. 10.1183/09041950.005s1693 24576915

[B48] Rechsteiner E. U. Rosen D. A. S. Trites A. W. (2013). Energy requirements of pacific white-sided dolphins (*Lagenorhynchus obliquidens*) as predicted by a bioenergetic model. *J. Mam.* 94 820–832. 10.1371/journal.pone.0105958 25162643 PMC4146581

[B49] Rojano-Doñate L. McDonald B. I. Wisniewska D. M. Johnson M. Teilmann J. Wahlberg M. (2018). High field metabolic rates of wild harbour porpoises. *J. Exp. Biol.* 221:jeb185827. 10.1242/jeb.185827 30523043

[B50] Scholander P. F. (1940). *Experimental Investigations on the Respiratory Function in Diving Mammals and Birds.* Oslo: I kommisjon hos Jacob Dybwad.

[B51] Sparling C. E. Fedak M. A. (2004). Metabolic rates of captive grey seals during voluntary diving. *J. Exp. Biol.* 207 1615–1624. 10.1242/jeb.00952 15073194

[B52] Thompson D. Fedak M. A. (1993). Cardiac responses of grey seals during diving at sea. *J. Exp. Biol.* 174 139–154. 8440964 10.1242/jeb.174.1.139

[B53] van der Hoop J. M. Fahlman A. Hurst T. Rocho-Levine J. Shorter A. K. Petrov V. (2014). Bottlenose dolphins modify behavior to reduce metabolic effect of tag attachment. *J. Exp. Biol.* 217 4229–4236. 10.1242/jeb.108225 25324344

[B54] Williams T. M. Friedl W. A. Haun J. E. (1993). The physiology of bottlenose dolphins (*Tursiops truncatus*): heart rate, metabolic rate and plasma lactate concentration during exercise. *J. Exp. Biol.* 179 31–46. 8340731 10.1242/jeb.179.1.31

[B55] Woakes A. J. Butler P. J. Bevan R. M. (1995). Implantable data logging system for heart rate and body temperature: its application to the estimation of field metabolic rates in antarctic predators. *Med. Biol. Eng. Comput.* 33 145–151. 10.1007/BF02523032 7643651

[B56] Worthy G. A. J. Worthy T. A. M. Yochem P. K. Dold C. (2013). Basal metabolism of an adult male killer whale (*Orcinus orca*). *Mar. Mam. Sci.* 30 1229–1237. 10.1242/jeb.137513 27385756

[B57] Yasuma F. Hayano J.-I. (2004). Respiratory sinus arrhythmia. *Chest* 125 683–690. 10.1378/chest.125.2.683 14769752

[B58] Yaw T. J. Kraus M. S. Ginsburg A. Clayton L. A. Hadfield C. A. Gelzer A. R. (2018). Comparison of a smartphone-based electrocardiogram device with standard six-lead electrocardiogram in the Atlantic bottlenose dolphin (*Tursiops truncatus*). *J. Zoo Wildlife Med.* 49 689–695. 10.1638/2017-0140.1 30212343

[B59] Yazdi P. Kilian A. Culik B. M. (1999). Energy expenditure of swimming bottlenose dolphins (*Tursiops truncatus*). *Mar. Biol.* 134 601–607. 10.1007/s002270050575 25324344

[B60] Yeates L. C. Houser D. S. (2008). Thermal tolerance in bottlenose dolphins (*Tursiops truncatus*). *J. Exp. Biol.* 211 3249–3257. 10.1242/jeb.020610 18840658

[B61] Young B. L. Rosen D. A. Hindle A. G. Haulena M. Trites A. W. (2011). Dive behaviour impacts the ability of heart rate to predict oxygen consumption in steller sea lions (*Eumetopias jubatus*) foraging at depth. *J. Exp. Biol.* 214 2267–2275. 10.1242/jeb.047340 21653820

[B62] Zena L. A. Leite C. A. C. Longhini L. S. Dias D. P. M. da Silva G. S. F. Hartzler L. K. (2017). Analysis of the respiratory component of heart rate variability in the cururu toad *Rhinella schneideri*. *Sci. Rep.* 7:16119. 10.1038/s41598-017-16350-0 29170531 PMC5701079

